# A Case of Successful Operation for Vesico-Vaginal Fistula

**Published:** 1854-11

**Authors:** 


### *A Case of Successful Operation for Vesico-Vaginal Fistula.*

*To the Editor of the N. Y. Medical Times:*

GENTLEMEN,—I beg leave, through the columns of your Journal, to make known to the medical profession, a case of *vesico-vaginal fistula*, successfully treated, according to the method of Dr. J. Marion Sims, of this city. This disease, which reduces the subject of it to a most deplorable condition, has hitherto been, to a very great extent, an opprobrium to surgical skill: very few surgeons, even in our large cities, have seen or known of a case of perfect cure.

To Dr. Sims is due the credit of having taken away this opprobrium. By the most patient and persevering experimentation, he has elaborated and perfected a method of suture, highly ingenious, and already sufficiently tested by experience to entitle it to rank among the great improvements of surgical art. It affords me peculiar pleasure to be able to contribute the following case, which may serve to strengthen the confidence of surgeons in the practical value of this operation.

As Dr. Sims has himself presented this subject to the profession within a few months, through other channels, I shall not enter into any detailed description of his peculiar method, farther than may be necessary to elucidate the case to be narrated.

Begging a favorable reception for this communication, I remain, very respectfully, Your ob't serv't,

GURDON BUCK, M.D., Surgeon to N. York Hospital.

CASE.—Mary Harty, an Irish woman, aged 28 years, married, of good constitution, and enjoying robust health, was admitted into Ward No. 2, in the New York Hospital, on the 4th July, 1854. About seven weeks previous, she was delivered of her first child after a very severe labor, but without the aid of instruments. It was soon discovered that she had lost all control over her bladder, and that her urine flowed from her constantly and involuntarily. This has continued to be her condition since; and from her own account, the flow of urine is not influenced by position. On examination, a fistula, capable of admitting the end of the little finger, was found in the median line, about one inch and three quarters beyond the meatus. It was situated in a transverse furrow, formed by the folding together of the vaginal walls. The parts in all other respects were healthy. On the 13th July, the operation was performed in the presence of several of the attending physicians and surgeons of the Hospital, and also of the assistants of the several divisions. The mode of procedure was as follows:

The patient's bowels having been previously evacuated with castoroil, an attempt was first made to operate with the patient placed upon her hands and knees, the buttocks being elevated as much as possible. This, however, was soon abandoned, from the impossibility of her maintaining herself in this position a sufficient length of time, and also, from its being inadmissible to employ ether in this position. She was therefore placed upon her back: and after being brought under the influence of ether, her hands and feet were bandaged together, as for lithotomy.

A Jacobson's lithotrite answered a most useful purpose in bringing within reach the fistulous opening, and keeping the surrounding vesico-vaginal wall on the stretch, thus very much facilitating the subsequent steps of the operation. This instrument resembles in form an ordinary catheter, with a short curve. The curve, when the instrument is opened, is converted into a jointed loop, or noose. It was introduced closed, per urethram into the bladder, and opened. By elevating the handle of the instrument toward the abdomen, the loop was depressed against the base of the bladder, forcing it downwards into the vagina, and forward towards the vulva. The fistula being brought thus within reach, its edges were pared away so as to render the longest diameter of the opening transverse. Six thread sutures were then introduced from before backwards; and by means of these, as many fine silver wires were drawn through to occupy their places. A narrow, flat strip of lead, called a clamp, perforated with slits at equal distances of a quarter of an inch apart, was threaded upon the four middle wires on either side of the wound, leaving one wire, at each end of the wound, free. Each of the four wires was then armed at both ends with a perforated shot, and the shot slipped down upon the clamps. A knot was also tied on the distal end of each wire. The proximal ends of the wires were then drawn forward till the distal clamp was brought to its place, in close contact with the posterior edge of the wound. The proximal clamp was next pushed backward to its place, and the shot with it; and so, both edges of the wound were held in close apposition between the two clamps. To secure them permanently in this position, the four shot were successively pinched tight upon the wires against the proximal clamp. The solitary wires at either end of the wound were armed with shot only, and secured in the same way as the wires passing through the clamps.

The adjustment of the sutures being completed, Dr. Sims' catheter was introduced and left in the bladder.

The patient was directed to lie upon her back; her diet was to consist of half a pint of milk and one soda cracker, three times a day. One single-grain pill of opium was to be taken morning and evening, to keep the bowels constipated. The catheter was to be removed and cleansed once in twenty-four hours, and replaced without delay.

The urine flowed in drops from the catheter, from the moment of its first introduction. No suppuration appeared at any time in the vagina, nor did the patient complain of much soreness in the region of the wound.

On the 20th of July, an evacuation of the bowels was produced by castor oil, followed by an enema of flax-seed tea.

On the 27th of July, the parts were examined for the first time, and union was found to have taken place throughout the entire wound. The anterior clamp had worn for itself a superficial furrow in the mucous membrane. The clamps and suture were removed, and no suppuration found.

The catheter was now left out for two hours, during which she remained dry. On being again introduced, the urine flowed in a jet.

The catheter was now left in the bladder only during the night, and resorted to at intervals of two hours, during the day.

On the 29th, the catheter was only introduced every three hours, and on the 31st, every four hours; the patient still keeping perfectly dry.

On the 1st of August, a careful examination was made, and every thing found in good condition. The abraded furrow made by the lower clamp had healed. Nearly five hours having elapsed since the catheter had been used, she was allowed for the first time to void her urine unassisted, which she did with a smart jet, and, as might be supposed, with the greatest delight. She now left her bed, and kept about. Up to the time of her discharge, on the 3d of August, the bladder performed its functions in a perfectly healthy manner, and the patient passed the night without rising.

On the 18th of August, the patient reported herself at the Hospital, still doing very well.—*New York Medical Times.*

